# Extremely elevated creatine kinase associated with rhabdomyolysis-induced acute kidney injury in a patient with Huntington’s disease: a case report

**DOI:** 10.1186/s13256-023-04018-5

**Published:** 2023-07-10

**Authors:** Isabelle Kozik, Zachary Wikerd

**Affiliations:** 1 Jacobs School of Medicine and Biomedical Sciences, University at Buffalo, Buffalo, NY 14203 USA; 2 Division of Geriatrics and Palliative Medicine, Department of Medicine, Jacobs School of Medicine and Biomedical Sciences, University at Buffalo, Buffalo, NY 14215 USA

**Keywords:** Rhabdomyolysis, Creatine kinase, Acute kidney injury, Huntington’s disease, Case report

## Abstract

**Background:**

Rhabdomyolysis-induced acute kidney injury is a serious condition that can progress to acute renal failure if not promptly identified and treated. Rhabdomyolysis occurs when serum creatine kinase levels approach > 1000 U/L (five times the normal upper limit). The chance of acute kidney injury increases as the levels of creatine kinase increase. Although Huntington’s disease is associated with muscle atrophy, elevated baseline creatine kinase levels in these patients have not been routinely reported.

**Case presentation:**

A 31-year-old African American patient presented to the emergency department after he was found unconscious from a fall attributed to the progression of his Huntington’s disease. On admission, he had an extremely high creatine kinase level of 114,400 U/L and was treated with fluids, electrolyte balance, and dialysis. However, his condition progressed to acute renal failure and he later developed posterior reversible encephalopathy syndrome, requiring transfer to the intensive care unit with placement on continuous renal replacement therapy. Eventually, his kidney function recovered and he was discharged home with 24/7 care by his family for persistent impairments related to his Huntington’s disease.

**Conclusions:**

This case report underscores the importance of promptly recognizing elevated creatine kinase levels in patients with Huntington’s disease due to the risk of developing rhabdomyolysis-induced acute kidney injury. If not aggressively treated, the condition of these patients is likely to progress to renal failure. Anticipating the progression of rhabdomyolysis-induced acute kidney injury is paramount to improving clinical outcomes. Additionally, this case identifies a potential link between the patient’s Huntington’s disease and his abnormally elevated creatine kinase, a finding not described in the literature of rhabdomyolysis-induced kidney injuries to date and an important consideration for future patients with similar comorbidities.

## Background

### Rhabdomyolysis

Rhabdomyolysis refers to the breakdown of skeletal muscle, which can result from a crush injury, strenuous exercise, infections, medications, substance abuse, metabolic/electrolyte disorders, and muscle hypoxia from artery occlusion, such as with compression from prolonged immobilization or during loss of consciousness (1). Rhabdomyolysis often presents clinically as limb weakness, myalgia, swelling, and grossly dark red- or brown-pigmented urine, which results from the high levels of myoglobin released from damaged muscle tissue (2).

The breakdown of muscle tissue can be detected as an increase in creatine kinase (CK) levels, and rhabdomyolysis is diagnosed when CK levels reach five times the normal range of 22–198 U/L. Damaged tissue also releases myoglobin, which can cause oxidative damage to kidney tubules and lead to acute kidney injury (AKI). AKI is a very serious complication of rhabdomyolysis that affects 10%–55% of patients (1, 3). If not identified early and treated appropriately, AKI can lead to fluid retention, electrolyte abnormalities, kidney failure, and death. The risk for AKI is greatly increased in patients with CK levels of ≥ 5000 U/L (4). We report a very unusual case, in which a patient presented to the emergency department with an AKI and a CK level > 500 times the normal limit.

### Huntington’s disease

Huntington’s disease is an autosomal dominant progressive neurodegenerative disorder characterized by > 35 CAG trinucleotide repeats in the gene that codes for the Huntingtin protein, which is essential for neuron function and is most concentrated in the brain (5). Continued deterioration of the striatal component of the basal ganglia and cortical neurons manifests as motor, cognitive, behavioral, and affective symptoms that ultimately lead to death within 20 years of symptom onset (5). Interestingly, though skeletal muscle wasting is a hallmark of Huntington’s disease, the mechanism underlying this atrophy is unknown, but is postulated to be caused by disrupted cell–cell signaling due to abnormal production of the Huntingtin protein (6). Motor manifestations of the disease, such as muscle rigidity and involuntary jerking movements, may contribute to muscle breakdown. Furthermore, sparse literature describes myopathy in Huntington’s disease in association with an abnormally elevated CK level. One case report described a semi-professional marathon runner with 43 CAG repeats who had a mildly elevated CK levels over years prior to the onset of chorea or other manifestations of Huntington’s disease (7). We describe a patient with Huntington’s with extremely elevated CK that leads to a rhabdomyolysis-induced AKI and consider his underlying disease as a predisposition for this abnormally high CK level.

## Case presentation

A 31-year-old African American male previously diagnosed with Huntington’s disease presented to a large public safety net hospital medical center after a family member found him immobilized on the ground at home. The patient was unable to determine how long he was down. In the emergency department, the patient was found to have an AKI with an elevated creatinine of 2.8 mg/dL and an extremely elevated CK level of 114,400 U/L. Medical records of the patient from three years prior showed a baseline glomerular filtration rate (GFR) of > 90 mL/min/1.73 m^2^ and a creatinine of 1.0 mg/dL. He also met criteria for systemic inflammatory response syndrome, with tachycardia, fever, and leukocytosis, and was therefore admitted to the hospital. A summary of the initial laboratory report is provided in Table 1.

**Table 1:** Laboratory values at time of admission

Category	Value	Reference range
Serum sodium (mmol/L)	136	133–145
Serum potassium (mmol/L)	4.3	3.3–5.1
Serum chloride (mmol/L)	100	96–108
Carbon dioxide (mmol/L)	22	19–30
Anion gap (mmol/L)	14	7–18
Blood urea nitrogen (mg/dL)	43	6–20
Creatinine (mg/dL)	2.8	0.7–1.2
Estimated glomerular filtration rate (mL/min/1.73 m^2^)	30	> 59
Lactic acid (mmol/L)	2.0	0.4–2.2
Calcium (mg/dL)	8.6	8.4–10.2
Phosphorus (mg/dL)	5.7	2.7–4.5
Creatinine kinase (U/L)	114,400	38–174
Total leukocyte count (cells/L)	14,300	4.8–10.8 (× 10^9^)
Hemoglobin (g/dL)	14.0	14.0–18.0
Platelets (cells/µL)	191,000	130,000–400,000

The patient presented with advanced Huntington’s disease characterized by dysarthria and choreiform movements of his hands. His family member who he lives with was able to provide additional history, reporting that the patient ambulates independently at home, though his gait is unsteady, and he had another fall 1 week prior. The patient performs his activities of daily living independently, but requires assistance with his instrumental activities of daily living, which family members provide for him. She further stated that several family members had passed away from Huntington’s disease and she had an understanding of the progressive degenerative nature of the disease.

Neurological exam showed an alert patient with equal and reactive pupils who was able to understand questions and responded with one word “yes” or “no” answers due to prominent dysarthria. Cranial nerves II–XII were intact. Muscle bulk was normal and muscle strength was 4/5 in the bilateral upper and lower extremities. Palpation of the muscles in the extremities did not elicit pain. On review of systems, the patient denied any muscle pain, joint aches, chest pain, or shortness of breath but reported head pain. A computed tomography scan of the head and cervical spine showed no signs of traumatic injury. A renal ultrasound showed bilateral renal parenchymal disease in the absence of stones, hydronephrosis, and obstruction. The results from blood cultures and urine toxicology were negative for abnormalities, though his urine was notably dark red-brown in color. Fractional excretion of sodium was 2%, consistent with an intrinsic renal injury.

Rhabdomyolysis-induced AKI was presumed, and the patient was treated initially with a 1 L bolus of normal saline followed by 200 mL/hour of lactated ringers for maintenance fluids. On day 4, the fluids were switched to 75 meq of sodium bicarbonate in 0.45% normal saline at 125 mL/hour due to metabolic acidosis. Electrolytes were repleted as needed. Over the next few days, the patient demonstrated progressive oliguria and showed signs of volume overload, noted by edema of the lower legs. Laboratory analyses of blood samples revealed progressive increases in the levels of creatinine and blood urea nitrogen (BUN) and a decrease in the glomerular filtration rate. CK levels continued to decrease from the peak level measured at admission. Due to worsening kidney function, with a creatinine of 13.9 mg/dL, the patient was dialyzed on day 7, day 9, and day 11 of his hospital stay. Over several days, gradual signs of renal recovery were observed, indicated by a down trending creatinine and BUN and an increasing GFR. A summary of the patient’s laboratory values over this time period are provided in Table 2.

**Table 2:** Select laboratory values from hospital days 5–12

Category	Day 5	Day 7	Day 9	Day 11	Day 12	Reference range
Creatinine (mg/dL)	12.2	13.9	10.9	9.7	7.2	0.7–1.2
Blood urea nitrogen (mg/dL)	111	125	80	60	37	6–20
Estimated GFR (mL/min/1.73 m^2^)	5	4	6	7	10	> 59
Anion gap (mmol/L)	19	20	15	16	16	7–18
Carbon dioxide (mmol/L)	24	21	21	23	25	19–30
Serum sodium (mmol/L)	140	141	140	141	139	133–145
Serum potassium (mmol/L)	4.5	4.5	4.6	4.4	4.6	3.3–5.1
Urine output (mL/day)	60	50	125	100	50	800–2000

On the 12th day of hospitalization, the patient became lethargic, hypoxic, and unresponsive. Because of the concern for a possible seizure, he was intubated for acute hypoxic respiratory failure and was transferred to the medical intensive care unit (ICU). Initial computed tomography (CT) imaging without contrast revealed ischemia, but follow-up magnetic resonance imaging without contrast revealed bilateral infarcts within a watershed distribution involving the frontal, parietal, and left occipital lobes of the brain, consistent with posterior reversible encephalopathy syndrome (PRES). Given that the patient had concurrent hypertension, oliguria, copious secretions present from his endotracheal tube, in addition to prior aggressive fluid treatment, volume overload was a likely cause of the PRES. On day 14, the patient was placed on continuous renal replacement therapy (CRRT) in place of hemodialysis due to his acute condition and worsening renal function with anuria. CRRT was initiated at a blood flow rate of 200 mL/minute and a dialysate rate of 1.5 L/hour. Over the subsequent days of treatment, these parameters ranged between blood flow rates of 200–300 mL/minute and dialysate rates of 1.5-3L/hour based on his electrolyte, BUN, and creatinine levels, as well as urine output. His ICU course was complicated by pneumonia and thrombosis of his central venous catheter, which interrupted CRRT from days 15–17 and required removal and replacement. On day 23, CRRT was stopped and hemodialysis was resumed 3 days per week, starting on day 24. On day 26, the patient was transferred back to the regular hospital floor. There was evidence of renal recovery indicated by a declining serum creatinine and a urine output of 800 mL on day 35.

Notably, the patient experienced marked dysphagia and episodes of vomiting even with a full liquid diet, requiring a nasogastric tube placement for nutrition on day 37. Subsequently, the patient underwent several food trials with the speech-language pathologist and was able to successfully complete them without concern for aspiration and the nasogastric tube was removed on day 44. He was evaluated by a physical therapist who arranged for an ambulation-assist device to be used at home. He was discharged home on day 46 with 24-hour care from his family. Labs upon discharge included a GFR of 63 mL/min/1.73 m^2^, creatinine of 1.5 mg/dL, and a BUN of 27 mg/dL.

The patient followed up as an outpatient with his primary care doctor two days after discharge. Neurologic exam showed intact cranial nerves II–XII, equal and reactive pupils, and continued dysphagia and unsteady gait. No new neurological deficits were documented. Unfortunately, medical records from the patient’s neurology clinic were inaccessible for review. Notably, the patient was readmitted to the same hospital for pneumonia in March of 2023 and was found to have return of baseline renal function with a GFR of 117 mL/min/1.73 m^2^, creatinine of 0.9 mg/dL, and a BUN of 6 mg/dL. Interestingly, his CK level was mildly elevated at 297 U/L, and in the absence of any other identifiable cause, supports him having an elevated level at baseline.

## Discussion

This case involved a patient with rhabdomyolysis in the setting of an extremely high CK level. Because this patient also had Huntington’s disease, we propose that comorbidities that impact skeletal muscle may increase baseline CK levels, and thus the risk for rhabdomyolysis-induced AKI. However, aggressive and effective treatment with fluids, electrolyte balance, and dialysis when needed can restore kidney function. Long-term survival among patients with rhabdomyolysis-induced AKI is near 80%, with renal function returning to baseline in most patients (8). It is critical to identify the presenting signs of AKI early and initiate prompt treatment to prevent permanent renal damage in patients with rhabdomyolysis.

A single-center study found that 65% (17/26) of patients with a CK level > 10,000 U/L, who also met the criteria for severe rhabdomyolysis, developed AKI (9). Furthermore, patients who developed acute renal failure had higher CK levels on admission, higher peak CK levels, and a slower decline of CK levels than patients who did not develop renal failure (9). The correlation between high CK levels and the incidence of AKI was confirmed in a meta-analysis (4). Thus, when CK levels are substantially elevated, as in our patient, it is essential to consider the possibility for severe kidney injury that may require dialysis.

The cause of rhabdomyolysis in the patient in the present case was likely compression of his limbs by his torso/head while he was immobile on the ground for a prolonged period after his fall. However, his extremely high CK level raises the question of whether another mechanism was a contributor, such as effects from progressing Huntington’s disease. Another case report described a semiprofessional marathon runner who was predisposed to Huntington’s disease (had 43 CAG repeats in the causative gene, *IT15*) in whom myopathy developed slowly and CK levels were elevated several years before symptoms of chorea were present (7). An examination of a biopsy sample from that patient revealed mild myopathy and mitochondrial dysfunction in skeletal muscle (7). These findings suggest that individuals with similar *IT15* variations are predisposed to elevated CK levels as a result of muscle breakdown.

Some limitations of this case include difficulty in obtaining an accurate initial history due to the patient’s fall being unwitnessed and the patient not being able to remember the incident in its entirety. Additionally, lack of the patient’s genetic information (number of CAG repeats) and time of his diagnosis limits our understanding of the severity of his disease. Strengths of this case include the ability to report a rare case of extremely elevated creatine kinase in a patient with Huntington’s disease, a rare disorder, something which is novel to the literature.

## Conclusion

Because of the strong association between CK and AKI, levels of CK should be monitored closely in patients with rhabdomyolysis. This may be particularly crucial for patients at risk for comorbidities impacting skeletal muscle integrity. We believe that the underlying Huntington’s disease-related atrophy of skeletal muscle in our patient increased the baseline levels of CK and contributed to the fall that precipitated and exacerbated the rhabdomyolysis-induced AKI.

## Data Availability

Not applicable.

## Abbreviations

CK: Creatinine kinase
AKI: Acute kidney injury
BUN: Blood urea nitrogen
GFR: Glomerular filtration rate
ICU: Intensive care unit
PRES: Posterior reversible encephalopathy syndrome
CRRT: Continuous renal replacement therapy
